# Hyponatremia among Postoperative Children Administered with Hypotonic Fluids in a Tertiary Care Hospital: A Descriptive Cross-sectional Study

**DOI:** 10.31729/jnma.7133

**Published:** 2021-11-30

**Authors:** Ashish Lal Shrestha, Susan Jehangir, Reju Joseph Thomas

**Affiliations:** 1 Department of Pediatric and Neonatal Surgery, Kathmandu Medical College and Teaching Hospital, Kathmandu, Nepal; 2 Department of Pediatric Surgery, Christian Medical College, Vellore 632 002 Tamilnadu, India

**Keywords:** *hyponatremia*, *hypotonic solutions*, *postoperative period*

## Abstract

**Introduction:**

Hypotonic solutions in postoperative children may cause hyponatremia. Considering humidity and temperatures in India, this study was conducted to find out the prevalence of hyponatremia among postoperative children who were administered with hypotonic solutions in a tertiary care hospital.

**Methods:**

A descriptive cross-sectional study was conducted at a tertiary care hospital. Ethical approval was taken from the institutional review board of Christian Medical College, Vellore, India (Reference number: 9177). Children aged less than 15 years undergoing elective surgery, requiring fasting for more than 12 hours post-operatively with normal preoperative electrolytes and renal functions were included. Hypotonic fluids were administered following existent protocol. Electrolytes were repeated immediate postoperatively and at 12-24 hours. Data was entered into and analyzed using the Statistical Package for the Social Sciences version 18.0. Point estimate at 90% Confidence Interval was calculated along with frequency and proportion for binary data.

**Results:**

Among 109 participants, hyponatremia in the postoperative period was seen in 53 (48.6%) (40.7-56.5 at 90% Confidence Interval) children. Hyponatremia was found in the immediate postoperative period in 10 (9.2%) children. All received Ringer Lactate as maintenance intra-operatively and none were severe enough to need correction. In the 12-24-hour sample, 43 (39.41%) had hyponatremia and none in severe category.

**Conclusions:**

Asymptomatic hyponatremia was noted in normal children planned for elective surgery. Among children managed with the existing institutional perioperative (hypotonic) fluid management protocol, subclinical postoperative hyponatremia within 12-24 hours of surgery was noted in a significant proportion, which was more in the hot and warm months in tropics. There are grounds for switching to isotonic fluids for perioperative management.

## INTRODUCTION

It has been a standard practice to administer hypotonic fluids as post operative alimentation in fasting children following major operations. Traditionally used fluids are either Iveolyte-P or N/2S+D5% based on patient's weight, both of which are constitutionally hypotonic in nature. This was based upon the recommendations of Holliday and Segar in their landmark paper published in 1957, popularly known as the 4-2-1 rule advocating the use of hypotonic fluids.^1,2^ It was later recognized to induce hyponatremia and serious associated complications.^3,4^

Post-operative hyponatremia, although earlier thought to be rare, has recently gained much attention among children receiving hypotonic intravenous fluids with serious consequences including neurological injury and death.5,6 Despite traditional practice, the necessity to revise and reevaluate it for Indian children is felt considering the tropical climate and insensible fluid losses in the form of sweating specially during hot and warm months.

This study was aimed at finding the prevalence of hyponatremia among postoperative children administered with hypotonic fluids in a tertiary care hospital.

## METHODS

This descriptive cross-sectional study was done in the Christian Medical College, Vellore, India from January 2015 to December 2016 among children less than 15 years of age with range of 11 days to 15 years. Ethical approval was taken from the Institutional Review Board (IRB) with the reference number IRB Min No: 9177. Convenience sampling was done and the sample size was calculated as,

n = Z^2^ × p × q / e^2^

  = (1.645)^2^ × 0.5 × 0.5 / (0.08)^2^

  = 106

Where,

n= required sample size,
Z= 1.645 at 90% Confidence Interval (CI),
p= prevalence taken as 50% for maximum sample size,
q= 1-p
e= margin of error, 8%

The calculated sample size was 106 but we included 109 children. Children who were to undergo elective surgery and needed to be fasted for at least 12 hours post operatively with normal preoperative serum electrolytes and renal functions were included in the study. Accordingly, all the selected patients had a baseline pre operative serum electrolyte done.

Children with underlying electrolyte abnormality (serum Na+ of <135 or >145mmol/l), renal disease, cardiac dysfunction, pre-existing hypertension, diuretic use, clinically detectable oedema, known adrenal dysfunction and neurological illnesses were excluded.

They were maintained on the standard intravenous fluid regimen N/2S+D5% or Iveolyte-P, both manufactured by Fresenius Kabi®) as per the current protocol accepted and in practice for children i.e.: For those > 10kg and or >1 year of age :- N/2S+D5%. For those < 10kg and or <1 year of age: - Buffered paediatric maintenance solution (Iveolyte-P) containing: Dextrose anhydrous: 5g, Potassium Chloride: 0.13g, Dibasic Potassium Phosphate: 0.026g, Sodium Acetate: 0.32g, Magnesium Chloride: 0.031g and Sodium Metabisulphite: 0.021g.

At the end of the procedure serum electrolytes was repeated. A second sample was sent 12-24 hours after the end of the procedure. Urine was collected and stored and sent only if serum sodium was abnormal i.e. <130mmol/l for urine osmolality, urine volume, urine spot sodium and urine specific gravity.

Hyponatremia was defined as serum Na+ <135mmol/l (reference laboratory value-Department of Biochemistry, Christian Medical College (CMC), Vellore, India: 135-145mmol/l). All the tests were analysed in the department of Clinical Biochemistry except for the Urinary specific gravity that was done in the department of Clinical Pathology following standard and conventional methods accepted worldwide. The information was readout from the Patients Results Page of the Clinical Workstation Software and steps were taken to correct hyponatremia according to established protocols.

In this manner the drop or any other alteration in the levels of serum sodium was studied. The results were analysed to see if postoperative hyponatremia (defined as Na+<135mmol/l) was a common occurrence in our perioperative management. Statistical analysis was done using the Statistical Package for the Social Sciences version 18.0. Point estimate at 90% CI was calculated along with frequency and percentage.

## RESULTS

Among 109 participants, hyponatremia in the postoperative period was seen in 53 (48.6%) children. In the immediate post operative period subclinical hyponatremia was noted in 10 (9.2%). In the 12-24-hour post operative period hyponatremia was found in 43 (39.41%) (Table 1).

**Table 1:** Serum Sodiumlevels inpre and postoperative periods (n=109).

		**Post operative**	
Serum Sodium	Preoperative n (%)	Immediate n (%)	12-24 hours n (%)
135-145	109 (100)	98 (89.9)	65 (59.6)
<135	0 (0)	10 (9.2)	43 (39.41)
>145	0 (0)	1 (0.9)	1 (0.9)

The most common operative elective procedure for which they were posted was open abdominal gastrointestinal surgery 86 (78.9%) (Table 2).

**Table 2:** List of Elective Procedures Requiring Post-Operative Fasting for 12 hours or more.

Type of Surgery	Systems		n (%)
1. Abdominal			
a. Open			
	Gastro Intestinal	86 (78.9)
	Urological	16 (14)
	Mixed Gastro Intestinal and Urological	5 (4.58)
b. Laparoscopic		1 (0.91)
2. Thoracic		1 (0.91)
	Total	109 (100)

Pre-operative blood level of serum electrolytes was done at mean duration of 9 days before the surgery (Ranging from same day to 5 weeks).

It was noted that the frequency of hyponatremia with Iveolyte-P was more than that with (N/2S+D5%) (Table 3).

**Table 3:** Prevalence of hyponatremia with use of Hypotonic Fluids.

Fluid type	Total	Na <135 mmol/L n (%)
Iveolyte-P	7	4 (57)
N/2S+D5%	102	39 (38.2)
Total	109	43

**Figure 1:**
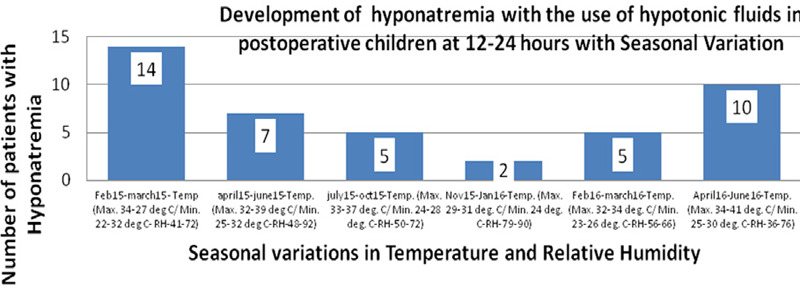
The variation in the prevalence of hyponatremia with month of the year.

The maximum cases occurred at temperatures of 31 35 Degree Celsius during warmer months (February-March/July-October) and hotter months (April-June) (Figure 1). The prevalence of hyponatremia was more during these months when relative humidity was also high.

There were 22 (20.2%) males and 21 (19.3%) females developing hyponatremia at 12-24 hours post operative period showing no particular gender predisposition.

The age range for those who developed hyponatremia in 12-24 hours post op period was found to be between 2.5 months-14 years.

No adverse event of clinical manifestations related to hyponatremia was noted.

## DISCUSSION

Of 131 children recruited in the study, one child had asymptomatic Hypernatremia (Na+ of 151mmol/l) with no identifiable cause. Hence, an incidental pre-operative hypernatremia with an prevalence of 0.7% (1/131) was noted. Similarly, asymptomatic hyponatremia of 16% (21/131) was also noted to exist preoperatively.

Of these, the possible cause could be predicted in 6/21 due to stoma related losses. Of these 3/21 had ileostomies, 2/21 had colostomies and 1 had a Malone's stoma on antegrade washes. However, in rest 15 the cause of hyponatremia could not be understood.

The prevalence of hyponatremia (mild) was 9% (10/109) in the immediate post operative period with Na+ range between 132-134mmol/l. In the second sample 12 to 24 hours after surgery, 39.4% (n= 43) had serum sodium value less than < 135mmol/l with range of Na+ between 129-134mmol/l.

In this group 97.6% (42/43) had mild and 2.3% (1/43) had moderate hyponatremia. No one had sodium levels below 125mmol/l and none had clinical manifestations of hyponatremia.

In this group of 43, with hyponatremia 4 had received Iveolyte-P and remaining 39/43 had received N/2S+D5%. Of those who did not develop hyponatremia, 3/66 had received Iveolyte-P and 63/66 had received N/2S+D5%.

Hyponatremia is defined as serum sodium level of <135mmol/l and has been classified by Joint European guidelines into mild-130-134mmol/l, moderate-125-129mmol/l and severe if <125mmol/l.5.^7-9^ There are several causes for the development of post operative hyponatremia. The over estimation of water requirement as is calculated using the 4-2-1 rule is considered the leading cause of hyponatremia. This formula that was originally meant for a well child in the awake state estimates the maintenance requirement of water by calorific expenditure, approximately 1 ml/cal.^1^ Based on this, standard pediatric maintenance fluids are hypotonic ie. they contain an excess of water over electrolytes and their osmolality is made up by the use of dextrose. Lindahl had found that energy expense during anesthesia was 50% lower than this and in fact close to basal metabolic rate.^10,11^

Acute hyponatremia, can also occur from excess release of Anti Diuretic Hormone (ADH). ADH could be released without osmotic causes in a post operative patient owing to the stimuli like stress, hemorrhage, opiates, pain, nausea and fever.^12,14^

Several recent articles caution the use of hypotonic fluids, if not strongly justifying the use of isotonic fluids, as safer practice. In 1992 Arieff had reported the incidence of post operative hyponatremia to be 0.34% and it had a mortality of 8.4%.^5^ In 2011 Choong had reported increased risk of hyponatremia with hypotonic fluids based on a study in 258 children.^15^ A recent meta-analysis by Wang, et al. in 2014 reported that the risks of hyponatremia was significant with use of hypotonic fluids based on 10 randomised controlled trials.^4^ No corresponding Indian study is available in literature. We wanted to study our patient population to see if we do have subclinical occurrence of hyponatremia and if we have, its cause in order to judge whether a change in practice is required.

Intra operative management of incurred deficits and third spacing along with intra-operative fluid management could influence changes in serum sodium levels. Hence to account for this serum sodium was estimated immediately after the surgery. In our study we found that in the immediate post operative period subclinical hyponatremia was noted in only 10 patients (9%).

In the 12-24 hour post operative period hyponatremia was found in 43 (39.41%). One child had a serum value of 129mmol/l, remained asymptomatic.

The strengths of our study are in being a prospective study and exclusion of those with preoperative hyponatremia. The weaknesses are from not using predetermined cohorts with age stratification and random allocation. The IV fluids were given following current unit practice.

## CONCLUSIONS

The study found significant subclinical hyponatremia when measured at 12-24 hours of surgery in children in whom hypotonic fluids were administered. The prevalence of hyponatremia was more in warm and hot months. Further evaluation is advised on the effects of isotonic fluids in practice.

The prevalence of hyponatremia was more in warm months (February-March/ July-October) and hot months (April-June) when the relative humidity was high. The maximum cases occurred at temperatures of 31-35 degree Celsius. The reason for this was probably owing to excessive sodium loss in sweat.

There was no correlation observed with the length of preoperative fasting or gender predisposition.

We identified four potential confounders in our study that could influence the results for example delay in recognising and replacing third spacing due to ileus; third spacing at the site of surgical injury; intravenous fluid boluses being given for hypotension, shock or poor urine output (bolus doses of isotonic fluid like normal saline is used and that could be an outcome modifier or may possibly be a confounder; dilutional agents used with drugs used in the postoperative period. Ileus and tissue third spacing would contribute to hyponatremia in the face of hypotonic fluid use. Back up of intestinal secretions and drainage by the nasogastric tube would be necessary for it to be recognised. Nasogastric tube drainage is replaced by 0.9% saline. However, the delay between the occurrence of ileus and replacement does give an opportunity for hyponatremia to occur. As per department protocol, 0.9% saline is used for fluid boluses in the face of hypotension or reduced urine output.

The reference value for Serum Na+ used in CMC, Vellore is based upon Tietz Fundamentals of Clinical Chemistry 6th Edition.^16^ The Nelson's textbook of pediatrics 20th Edition gives normal serum Na+ reference range as 135-145mmol/l for pediatric age group.^17^ We found the range of 130-151 mmol/l before surgery in clinically asymptomatic patients.
